# PRKCD Orchestrates Sevoflurane‐Induced Cognitive Dysfunction in Aged Rats by Regulating PINK1/PRKN Mitophagy Pathway

**DOI:** 10.1111/jcmm.71315

**Published:** 2026-08-10

**Authors:** Yang Yu, Wei Zhang, Yingying Zhao, Yue Zhang, Pingle Li, Zepeng He

**Affiliations:** ^1^ Department of Anesthesiology Pain and Perioperative Medicine, the First Affiliated Hospital of Zhengzhou University Zhengzhou Henan China

**Keywords:** mitophagy, post‐operative cognitive dysfunction, PRKCD, PRKN, sevoflurane

## Abstract

Post‐operative cognitive dysfunction (POCD) is a cognitive disorder characterized by a decline in cognitive function following surgical procedures, with mitophagy identified as a significant underlying mechanism. Protein kinase C delta (PRKCD), localized within the mitochondria, is implicated in the regulation of PINK1/PRKN mitophagy pathway; however, the potential regulatory role of PRKCD in POCD through this pathway remains to be elucidated. Neurons and rats were exposed to sevoflurane (SEV) to illuminate the function and mechanism of PRKCD in POCD. Various methodologies were employed, including immunofluorescence, quantitative real‐time PCR, CCK‐8 assays, mitochondrial membrane potential (MMP) assessments, MitoSOX generation detection, Seahorse metabolic flux analysis, co‐immunoprecipitation, western blotting and behavioural experiments like Morris water maze, novel object recognition and fear conditioning, along with haematoxylin and eosin and immunohistochemical staining. PRKCD was expressed in neurons and that SEV administration led to an upregulation of PRKCD expression. Furthermore, interference with PRKCD was found to restore cell viability in SEV‐treated neurons. Additionally, inhibition of PRKCD resulted in the recovery of LC3 expression and the normalization of p62 levels in neurons subjected to SEV treatment. Suppressing PRKCD restored MMP and OCR, reduced MitoSOX in SEV‐affected neurons and interacted with PRKN and PINK1, decreasing their expression. Overexpressing PRKN mitigated PRKCD inhibition's impact on mitochondrial damage. In vivo, SEV increased PRKCD, PINK1 and PRKN levels, but PRKCD knockdown improved behavioural and pathological outcomes, reversing changes in LC3‐II, PINK1, PRKN and p62 expression. PRKCD enhanced SEV‐induced POCD in aged rats via the regulation of PINK1/PRKN mitophagy pathway.

## 1 Introduction

Post‐operative cognitive dysfunction (POCD) is a cognitive disorder that arises following anaesthesia. POCD patients have impaired memory, attention, information processing ability, visuospatial ability and other cognitive abilities [1]. The POCD incidence ranges from 10% to 65%, especially in the elderly [2], with 9% to 46% incidence in surgical patients over the age of 65 [3]. Another study shows that with improvements in overall living standards, healthcare, nutrition and education, elderly patients now make up an increasing proportion of the surgical population, while the POCD incidence in people over 60 years old is at least twice as high as that in younger people [4]. POCD patients require longer hospital stays, and cognitive deficits can have devastating consequences on patients' capability to continue in the labour market and abide independent of social transfer payments, which not only brings additional burden to patients' families, but also greatly affects patients' quality of life [5, 6]. POCD has become an urgent health and social problem to be solved, especially in elderly patients [7]. Therefore, in‐depth research on POCD is the premise of prevention and treatment of POCD, which has important medical and social value.

Mitochondria are among the most intricate and vital organelles found in eukaryotic cells, responsible for the conversion of organic substrates into carbon dioxide and water via redox reactions [8]. Breakdown of mitochondria can disrupt the proper transfer of electrons, begotten during these redox processes, to oxygen, water, or their intermediary states, resulting in the excessive creation of reactive oxygen species (ROS), which may lastly lead to cellular apoptosis [9, 10]. Mitophagy, a specialized form of autophagy, involves the selective degradation of surplus or damaged mitochondria, thereby playing a decisive role in the subsistence of mitochondrial homeostasis and the management of mitochondrial population within cells [11, 12]. Under various stress conditions, mitophagy can be activated and enhanced mitophagy has been shown to promote cellular survival by eliminating dysfunctional mitochondria [13]. Current studies have increasingly highlighted the value of mitophagy induction in the prevention of POCD [14].

Investigations into the molecular mechanisms underlying mitophagy have predominantly concentrated on hereditary forms of Parkinson's disease (PD) and the roles of the genes *PINK1* and *Parkin* (*PRKN*) in mediating mitophagy in terms of mitochondrial depolarization [15]. Research has demonstrated that the choosy identification of defective mitochondria is mediated by PINK1‐dependent phosphorylation of ubiquitin and PRKN, which subsequently leads to the ubiquitination of outer mitochondrial membrane proteins. These proteins are then recognized through specified autophagy receptors that connect with LC3 and GABARAP proteins on the autophagic membrane, facilitating the organization of mitophagosomes. While the function of PRKN has been associated with the management of stress‐evoked mitophagy in vivo, as observed in conditions such as myocardial infarction [16] and alcohol‐induced liver disease [17], basal mitophagy seems to occur chiefly independently of the PINK1/PRKN pathway. One alternative pathway involves the hypoxia‐inducible factor 1‐alpha (HIF1α), which is upregulated under hypoxic conditions and enhances the expression of the mitophagy receptor BNIP3, a protein notably characterized for its function in the liquidation of mitochondria from red blood cells [18]. Furthermore, diverse small molecules, like cobalt chloride, dimethyloxallyl glycine and iron chelator deferiprone [19, 20], have been identified to stabilize HIF1α and replicate a hypoxia‐evoked mitophagy phenotype without necessitating hypoxic status [21]. A recent investigation has demonstrated that cyclin G‐associated kinase (GAK) and protein kinase C delta (PRKCD) specifically modulate HIF1α‐dependent mitophagy without influencing PRKN‐dependent mitophagy, and these kinases also play a role in regulating basal mitophagy in vivo [22].

PRKCD is a part of the extensive PKC kinase family and is classified within the subgroup of novel PKCs (nPKCs), which includes PRKCE, PRKCH and PRKCQ, all of which are activated individually of Ca^2+^ by diacylglycerol [23]. PRKCD has been shown to regulate cell proliferation, the cell cycle and apoptosis, primarily through the mediation of target protein phosphorylation [24]. Consequently, PRKCD has been entangled in disparate diseases, like cancers [25, 26], cardiovascular diseases [27], type 2 diabetes mellitus [28], celiac disease [29] and hemiplegic cerebral palsy [30]. Furthermore, prior research indicates that PRKCD expression is upregulated in mice exposed to sevoflurane (SEV), and the PRKCD knockout mitigates SEV‐elicited neurotoxicity and cognitive impairment in mice [31]. Nonetheless, it remains unclear whether PRKCD regulates POCD through mitophagy that is independent or dependent on the PINK1/PRKN axis.

In this study, we demonstrated that the inhibition of PRKCD can mitigate mitochondrial damage and enhance mitophagy, thereby alleviating SEV‐induced POCD. We subsequently proposed and validated a mechanism of PRKCD‐mediated regulation of PINK1/PRKN mitophagy pathway that ameliorated SEV‐induced POCD.

## 2 Materials and Methods

### 2.1 Animals

Seventy‐five healthy male Sprague–Dawley rats, aged 18 months and weighing between 550 and 650 g, were procured from the Experimental Animal Centre of our university. All animals were housed in a temperature‐controlled environment with unrestricted access to food and water. All experimental procedures involving animals were conducted in accordance with the guidelines set forth by the Animal Ethics and Welfare Committee of our university as well as the Guide for the Care and Use of Laboratory Animals [31].

### 2.2 Primary Hippocampal Neuron Isolation

Hippocampal neurons were isolated from neonatal Sprague–Dawley rats using a method [32]. Neonatal rats were euthanized with carbon dioxide, and then the brain was dissected. Following the removal of the meninges, hippocampal tissues were collected and the neurons were isolated involving digestion with trypsin (catalogue numbers: T8150, Solarbio, Beijing, China) and DNase I (catalogue numbers: D8070, Solarbio) in HBSS (catalogue numbers: H1147, Solarbio) at 37°C for 5 min. The assimilation was halted with Neurobasal medium (catalogue numbers: 21103–049, Gibco, Rockville, MD, USA) containing B27 (catalogue numbers: B3075, Solarbio), Penicillin–Streptomycin (catalogue numbers: P1400, Solarbio), L‐glutamine (catalogue numbers: G0200, Solarbio) and FBS (catalogue numbers: S9000, Solarbio). After a 10‐min incubation at 37°C, cells were centrifuged, resuspended, mechanically dissociated, filtered and seeded onto PDL‐coated plates (catalogue numbers: 354470, Corning Company, New York, NY, USA). The medium was switched to serum‐free B27 Neurobasal following 24 h, and cultures were retained at 37°C with 5% CO_2_ for 8–9 days prior to use.

### 2.3 Cortical Glial Culture Operations

Mixed glial cell cultures were derived from the cortical lobes of newborn Sprague–Dawley rats as in earlier research [33]. The lobes were treated with trypsin and DNase I, then digestion was halted with IMDM (catalogue numbers: C3310, Solarbio) containing antibiotics and FBS. Cells were centrifuged, resuspended and mechanically dissociated before being plated in PDL‐coated flasks. The medium was changed the next day to either IMDM for astrocytes or DMEM (catalogue numbers: 31600, Solarbio) for microglia. Astrocytes were harvested after 1 week, and microglia after 2 weeks, both retained at 37°C with 5% CO_2_. Primary astrocytes and microglia were isolated from glial mixtures. Astrocytes were trypsinized, centrifuged and plated on PDL‐coated plates, with the medium switched to serum‐free B27 Neurobasal after 24 h. Microglia were collected by orbital shaking, centrifuged and plated similarly. Both cell types were cultivated at 37°C, 5% CO_2_.

### 2.4 Immunofluorescence

Neurons and glial cells were seeded onto glass plates and cultivated at 37°C, 5% CO_2_. Cells were managed with 4% paraformaldehyde (catalogue numbers: P1110, Solarbio) at room temperature for 15 min, bovine serum albumin blocking buffer (catalogue numbers: SW3015, Solarbio) overnight at 4°C and 0.2% Triton X‐100 (catalogue numbers: T8200, Solarbio), along with primary antibodies. The primary antibodies were anti‐MAP2 (1:1600, catalogue numbers: PA5‐17646, RRIDs: AB_11006358, Invitrogen, Carlsbad, CA, USA), anti‐Iba1 (1:1000, catalogue numbers: PA5‐27436, RRIDs: AB_2544912, Invitrogen) and anti‐GFAP (1:250, catalogue numbers: PA5‐16291, RRIDs: AB_10980769, Invitrogen). Subsequently, cells were gestated with Goat Anti‐Rabbit IgG H&L (Alexa Fluor 488) (1:1000, catalogue numbers: ab150077, RRIDs: AB_2630356, Abcam, Cambridge, UK) for 1 h at room temperature. Last, both the cell cultures and tissue sections were stained with an antifading mounting medium containing DAPI (catalogue numbers: S2110, Solarbio) and visualized via fluorescence microscopy (IX71, Olympus, Tokyo, Japan).

### 2.5 Quantitative Real‐Time PCR (qRT‐PCR)

Total RNAs were obtained from cells through TRIzol (catalogue numbers: 15596026, Solarbio). Afterward, cDNA for mRNA was synthesized with PrimeScript RT Master Mix (catalogue numbers: RR037A, TaKaRa, Tokyo, Japan) and qRT‐PCR was implemented with SYBR Green I (catalogue numbers: SY1020, Solarbio) on an ABI 7900 system (Applied Biosystems, Foster, USA). Relative mRNA levels were normalized to GAPDH by 2^−ΔΔCt^. The primer sequences were: 5′‐ACCTCTTCTTTGTGATGGAGTTCC‐3′ (PRKCD forward), 5′‐AGCTTGAGGTCCCTGTAAATGATG‐3′ (PRKCD reverse); 5′‐ACGGCAAGTTCAACGGCACAGTCA‐3′ (GAPDH forward), 5′‐CCACGACATACTCAGCACCAGCATCA‐3′ (GAPDH reverse).

### 2.6 Cell Treatment and Grouping

Hippocampal neurons were indiscriminately delegated to 5 groups: control, SEV (4.1% SEV for 6 h [31]), SEV + Rottlerin (treated with 1 μM Rottlerin for 30 min [34]), SEV + siPRKCD (managed with 50 nM siPRKCD for 24 h and 4.1% SEV for 6 h) and SEV + siPRKCD+PRKN group (administrated with 50 nM siPRKCD and PRKN overexpression plasmids for 24 h after 4.1% SEV for 6 h). All the siRNAs were designed and provided by GenePharma (Shanghai, China).

### 2.7 Western Blotting

Followed by cells and tissues were lysed in RIPA (catalogue numbers: R0010, Solarbio), protein concentrations were quantified with BCA Protein Colorimetric Assay Kits (catalogue numbers: E‐BC‐K318‐M, Elabscience, Wuhan, China). Proteins were further separated with 10% SDS‐PAGE (catalogue numbers: BT041215BOX, Thermo Fisher Scientific, Waltham, MA, USA). Next, proteins were electroblotted onto PVDF membranes (catalogue numbers: IPVH00010, Millipore, Billerica, USA) and blocked in 5% skim milk (catalogue numbers: D8340, Solarbio) for 1.5 h. Membranes were probed with primary antibodies against PRKCD (catalogue numbers: PA5‐87443, 1:2000, RRIDs: AB_2804152, Invitrogen), LC3‐I/II (catalogue numbers: PA1‐16931, RRIDs: AB_2137583, 1:1000, Invitrogen), p62 (catalogue numbers: PA5‐20839, RRIDs: AB_11157045, 1:1000, Invitrogen), PINK1 (catalogue numbers: PA1‐16604, RRIDs: AB_2164267, 1:1000, Invitrogen), PRKN (catalogue numbers: 740019R, RRIDs: AB_3074189, 1:1000, Invitrogen) and GAPDH (catalogue numbers: ab8245, RRIDs: AB_2107448, 1:2000, Invitrogen) overnight at 4°C. Membranes were further exposed to horseradish peroxidase‐conjugated secondary antibodies (catalogue numbers: ab205719, RRIDs: AB_2755049, 1:2000, Abcam). Protein bands were visualized using the ECL Kits (catalogue numbers: PE0010, Solarbio) and Quantity One Software (Bio‐Rad, Hercules, CA, USA).

### 2.8 Cell Counting Kit‐8

To examine cell viability, neurons (5 × 10^3^) were sourced in 96‐well plates overnight. Soon afterwards, CCK‐8 solution (10 μL, catalogue numbers: CA1210, Solarbio) was mixed and cultured for further 4 h in accordance with manufacturer's protocol. Optical density (OD) values at 450 nm were obtained using a microplate reader (Bio‐Rad).

### 2.9 Detection of Mitochondrial Membrane Potential (MMP)

The MMP was examined via the Mitochondrial Membrane Potential Assay Kit with JC‐1 (catalogue numbers: M8650, Solarbio). In brief, post‐administration, cells were collected and incubated with 1 mL of culture medium containing JC‐1 fluorescent dye for 20 min at 37°C. Subsequently, cells were centrifuged at 600 × g for 5 min at 4°C, and the sedimented cells were resuspended in 1 × JC‐1 buffer. Five fields were randomly chosen for the analysis.

### 2.10 Detection of MitoSOX Generation

MitoSOX was detected using the MitoSOX Red dye from Yeasen Biotechnology (catalogue numbers: 40778ES50, Shanghai, China). Cells were cultivated in 6‐well plates overnight and managed as specified. For flow cytometry analysis, cells were collected and processed by the FACSCalibur instrument instructions, with results shown as a histogram of mean MitoSOX fluorescence intensity.

### 2.11 Seahorse Metabolic Flux Analysis

The oxygen consumption rate (OCR) was appraised through the Seahorse XF 96 Analyser and the Seahorse XF Cell Mito Stress Test kit (catalogue numbers: 103015–100, Agilent Technologies, Shanghai, China). Cells (2 × 10^4^ per well) were seeded in a 96‐well plate, and the sensor cartridge was calibrated. Oligomycin (1.5 μM, catalogue numbers: 08150, Solarbio), FCCP (1.5 μM, catalogue numbers: IF06901, Solarbio), rotenone and antimycin A (1.25 μM, catalogue numbers: A7680, Solarbio) were added sequentially at 20, 40 and 60 min. OCR data were analysed with the Seahorse XF‐96 Wave software version 2.6.

### 2.12 Co‐Immunoprecipitation (Co‐IP) Experiment

Cells were lysed using a lysis buffer (catalogue numbers: P1206, Solarbio) supplemented with protease inhibitors (Solarbio) for 40 min on ice to garner the supernatant. To investigate the interaction between PRKCD and PRKN as well as PINK1, antibodies against PRKCD (catalogue numbers: PA5‐17552, RRIDs: AB_10985479, Invitrogen), PRKN (catalogue numbers: 740019R, RRIDs: AB_3074189, Invitrogen), PINK1 (catalogue numbers: 23274–1‐AP, RRIDs: AB_2879244, Proteintech, Chicago, USA), or immunoglobulin G (anti‐IgG) (catalogue numbers: ab172730, RRIDs: AB_2687931, Abcam) were mixed to the supernatant and bred at 4°C overnight. Subsequently, protein A/G‐agarose beads (catalogue numbers: PI20421, Thermo Fisher Scientific) were introduced and agitated at 4°C. The resulting cell pellets were collected and washed with lysis buffer. The precipitates were then boiled with loading buffer (catalogue numbers: R1055, Solarbio) for 5 min and analysed through western blotting.

### 2.13 Animal Model Preparation

Animals were assigned to experimental groups using a random number table–based randomization procedure. Two rats were housed per cage. Behavioural testing and scoring were performed by an experimenter blinded to group allocation. Tissue collection and subsequent molecular analyses were conducted by a second investigator who was also blinded to group assignment. The humane endpoints were daily assessed according to the ARRIVE guidelines and rats reaching humane endpoints were euthanized by induction anaesthesia with intraperitoneal injections of sodium pentobarbital and cervical dislocation. In this study, all rats were used as the data included in the experimental and control data, and there were no exclusions. All animals were housed in a temperature‐controlled environment with unrestricted access to food and water and handled by experienced personnel. All efforts were made to minimize both the suffering and the number of animals.

After one week of acclimatization, the animal experiments were carried in two phases. In the preliminary phase, rats were arbitrarily attributed to receive either sham or SEV exposure (*n* = 6 per group). Rats were positioned in an acrylic chamber connected to a sevoflurane vaporizer (Drager, Germany) and a multi‐gas monitor (Datex‐Ohmeda, USA). SEV‐exposed rats received 2.6% sevoflurane in humidified 30% O_2_ for 4 h, while sham controls received humidified 30% O_2_ balanced with N_2_ for the same duration. Rectal temperature was maintained at 37°C ± 0.5°C throughout the procedure. Hippocampal tissues were collected at 7, 14 and 28 days post‐exposure to determine the temporal profile of PRKCD, PINK1 and PRKN expression.

Based on the preliminary results, the main experiments were conducted using the 7‐day post‐exposure time point. Rats were randomly divided into three groups (*n* = 10 per group): sham, SEV and SEV + AAV‐shPRKCD. All three groups received bilateral intrahippocampal injection of AAV at the following coordinates relative to bregma: anteroposterior −3.3 mm, mediolateral ±2.0 mm, dorsoventral −2.8 mm. The sham and SEV groups received AAV expressing scrambled shRNA (5 × 10^12 viral genomes/mL, 2 μL per side), while the SEV + AAV‐shPRKCD group received AAV expressing shRNA targeting PRKCD at the same titre and volume. Fourteen days after AAV injection, rats in the SEV and SEV + AAV‐shPRKCD groups received a single 4‐h exposure to 2.6% sevoflurane based on the previous studies [35, 36], whereas sham‐group rats received humidified 30% O_2_ balanced with N_2_ for the same duration. Behavioural tests were performed 3–7 days post‐exposure, and rats were sacrificed on Day 7 for tissue collection.

### 2.14 Morris Water Maze (MWM) Test

The MWM test was conducted 3–7 days after sevoflurane exposure to gauge spatial learning and memory. The apparatus consisted of a circular pool (150 cm diameter, 50 cm depth) filled with water maintained at 22°C ± 1°C. The water was made opaque by adding non‐toxic white paint. A circular escape platform (10 cm diameter) was submerged 1 cm below the water surface in the centre of the target quadrant. Visual cues were placed on the walls surrounding the pool to provide spatial references.

During the acquisition phase (days 3–6 post‐exposure), rats received four training trials per day with an inter‐trial interval of 30 min. For each trial, rats were installed into the water facing the pool wall at one of four designated starting positions in a pseudorandom order. Rats were allowed up to 60 s to locate the hidden platform. If a rat failed to seek the platform within 60 s, it was gently guided to the platform and allowed to remain there for 15 s before being returned to its home cage. Escape latency, defined as the time required to reach the platform, was recorded for each trial.

On Day 7 post‐exposure, a probe trial was governed to assess spatial memory. The platform was removed from the pool, and rats were allowed to swim freely for 60 s. The number of platform location crossings, time spent in the target quadrant, and swimming path length were recorded and analysed using a computerized video tracking system.

### 2.15 Novel Object Recognition Test

This test assessed learning and memory in rats, leveraging their natural curiosity for new objects. The setup included a rectangular box and three objects (A, B and C), with A and B being identical and C distinct. The dimensions of the rectangular box were 72 cm × 72 cm × 50 cm. The dimensions of the two familiar objects (A and B) were 8 cm in height and 4 cm in diameter. The dimensions of the novel object (C) were 5 cm in height, 6 cm in length and 4 cm in width. Over the first two days, rats (*n* = 10) acclimated to the area for 10 min. On the third day, the rat explored objects A and B in the box for 5 min, then returned to its cage. After 1 h, object B was replaced by C, and the rat explored again for 5 min. The area and objects were cleaned with ethanol. The recognition index was computed by dividing the time spent on the novel object by the total exploration time.

### 2.16 Fear Conditioning Test

In this test, rats were placed in a training box for 3 min, then exposed to two cycles of a 30‐s tone (75 dB, 1000 Hz) paired with a 0.8 mA foot‐shock in the last 2 s, with 60‐s intervals between cycles. After 60 s post‐final shock, they returned to their cages. Contextual and tone fear memory was assessed 24 h later. For the contextual test, rats were situated in the training box for 5 min without stimuli, with freezing indicating learned association. In the tone test, conducted 2 h later, rats adapted for 3 min in a test box before hearing the tone for 30 s, concluding the experiment 1 min later. Following each test, the chamber was sanitized with 75% alcohol to remove any olfactory cues. Freezing behaviour was characterized by the absence of any visible movement except for respiration and was checked using Tracking Master V3.0.

### 2.17 Haematoxylin and Eosin (H&E) Staining

The tissue samples underwent standard pathological staining procedures. Sections of 5 μm thickness were stained with haematoxylin and eosin (H&E) (catalogue numbers: G1120, Solarbio) and thereafter analysed with light microscopy (BX53, Olympus) and image analysis software Image‐Pro Plus 6.0 (Media Cybernetics, USA).

### 2.18 Immunohistochemistry

Paraffin‐coated tissues were restored with sodium citrate buffer at 94°C for 15 min, sealed with 1% BSA (catalogue numbers: SW3015, Solarbio) for an hour, and brooded with primary antibodies against PRKCD (catalogue numbers: PA5‐17552, RRIDs: AB_10985479, Invitrogen) overnight at 4°C. A secondary HRP‐labelled anti‐rabbit IgG antibody (catalogue numbers: 31460, RRIDs: AB_228341, Invitrogen) was applied at 37°C for 30 min. The slices were re‐stained with haematoxylin (catalogue numbers: SH8390, Solarbio), and imaged via a light microscope (BX53, Olympus).

### 2.19 Statistical Analysis

Data were introduced as mean ± standard deviation (SD) and were assayed using the SPSS 20.0 software (IBM, Armonk, New York, USA). Statistical significance was resolved using the Student's *t*‐test for comparisons between two groups, or one‐way analysis of variance (ANOVA) followed by the Bonferroni post hoc test for comparisons involving more than two groups. Parametric statistical tests were applied throughout. Given the small sample sizes employed in in vitro experiments (*n* = 3 per condition), formal normality testing (e.g., Shapiro–Wilk) was not performed, as such tests have insufficient statistical power to reliably detect non‐normality at this sample size [37]. Parametric analyses were considered appropriate given the continuous nature of the outcome variables and their consistency with standard practice in comparable published studies. The absence of formal normality and variance homogeneity assessment was acknowledged as a limitation of this study. For the Morris water maze acquisition phase, escape latency data were analysed using two‐way repeated‐measures analysis of variance (ANOVA), with group as the between‐subject factor and training day as the within‐subject repeated factor. Sphericity was assessed using Mauchly's test, and the Greenhouse–Geisser correction was applied when the sphericity assumption was violated. Post hoc pairwise comparisons were conducted with Bonferroni adjustment. *p* < 0.05 was defined as significant difference. No formal a priori sample size calculation was performed; sample sizes were determined based on previously published studies employing similar experimental paradigms [38, 39, 40, 41, 42]. The absence of a power analysis represented a limitation of this study.

## 3 Results

### 3.1 PRKCD Was Upregulated in SEV‐Induced Neurons

**FIGURE 1:**
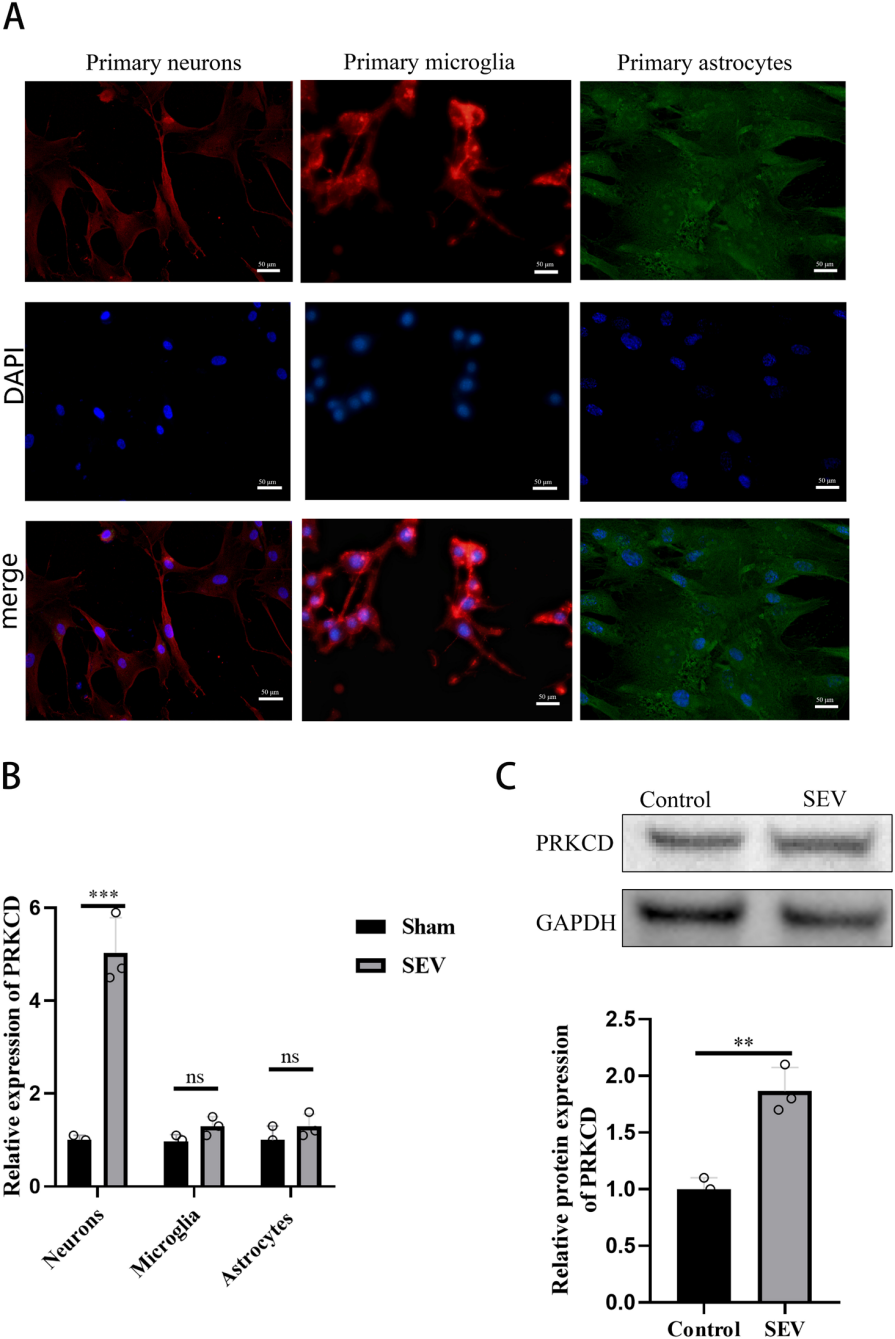
An upregulation of PRKCD in SEV‐induced neurons. (A) Identification of separated neurons, astrocytes and microglia from rats by immunofluorescence using MAP2, Iba1 and GFAP antibodies. (B) Transcriptional levels of PRKCD in separated neurons, astrocytes and microglia. (C) Translational expressions of PRKCD in SEV‐induced neurons. *N* = 3.

The PRKCD level was first examined in the separated neurons, astrocytes and microglia from rats (Figure 1A), and the results showed that PRKCD was expressed in neurons, but not in microglia or astrocytes (Figure 1B). To explore the PRKCD role in POCD, neurons were treated with SEV, and an increased expression of PRKCD was observed (Figure 1C). Thus, PRKCD expression was elevated in SEV‐evoked neurons.

### 3.2 The Indicative Role of PRKCD Inhibition in the Improved Mitophagic Clearance in SEV‐Induced Neurons

**FIGURE 2:**
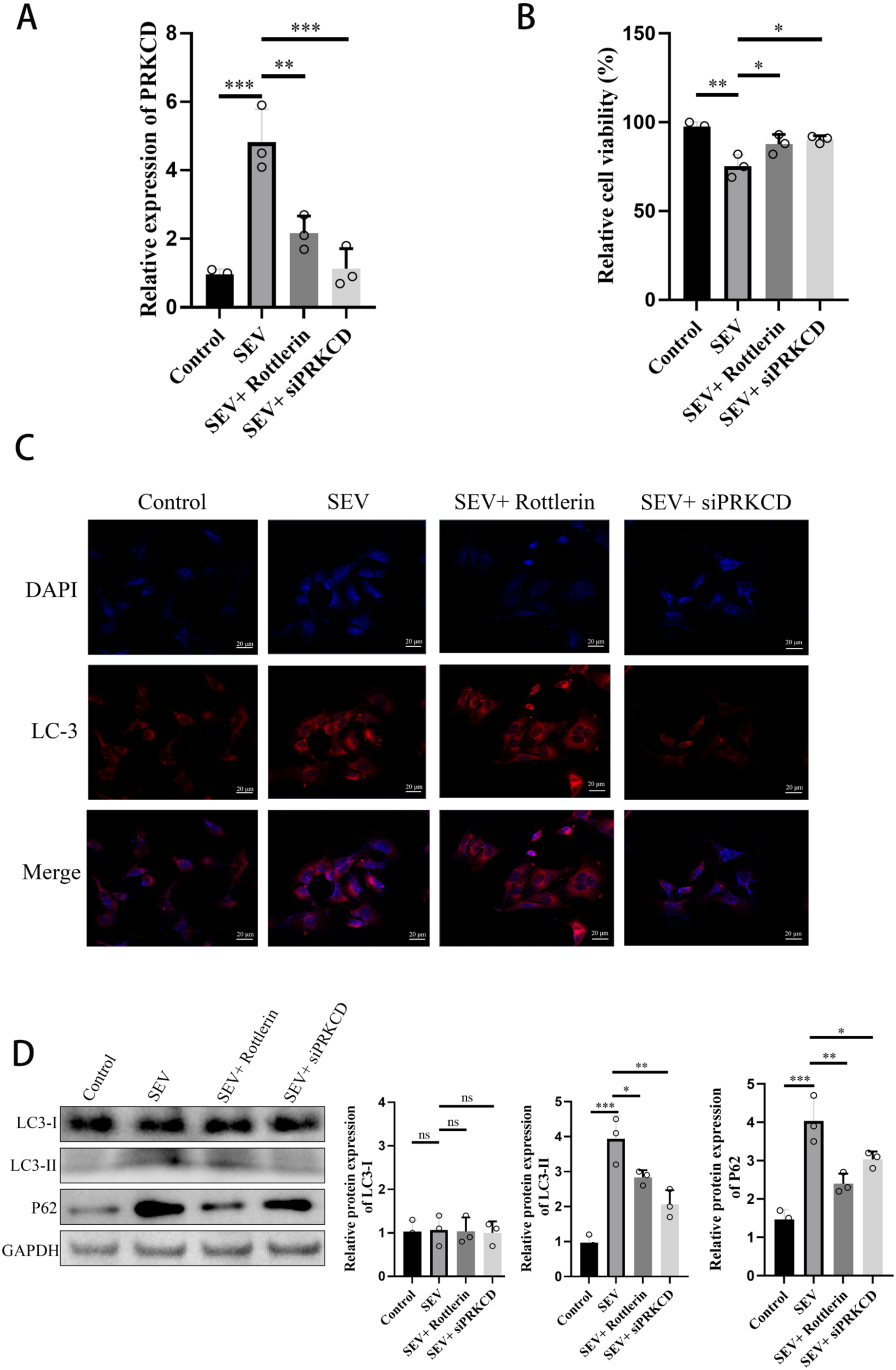
PRKCD interference accelerated mitophagy in SEV‐evoked neurons. Neurons were treated with 4.1% SEV for 6 h, and then incubated with the inhibitor of PRKCD, rottlerin for 24 h. Besides, siPRKCD was transfected into neurons and then administrated with 4.1% SEV for 6 h. (A) Transcriptional levels of PRKCD by RT‐qPCR. (B) Cell viability by CCK‐8. (C) LC‐3 expression by immunofluorescence. (D) Expressions of LC3‐I, LC3‐II and p62 by western blot. *N* = 3.

To inspect the PRKCD role in neuronal mitophagy, neurons were treated with the PRKCD inhibitor rottlerin or transfected with PRKCD‐specific siRNA before SEV exposure (Figure 2A). Cell viability assays showed that both PRKCD inhibition approaches reversed the SEV‐induced reduction in neuronal survival (Figure 2B). This study assessed mitophagic flux by examining LC3 and p62 protein levels. Immunofluorescence staining revealed increased LC3 puncta in neurons treated with rottlerin or PRKCD siRNA compared to SEV alone (Figure 2C). Western blot analysis showed that SEV exposure increased both the LC3‐II/LC3‐I ratio and p62 expression (Figure 2D). The concurrent increase in LC3‐II and p62 indicates impaired mitophagic flux. Both rottlerin treatment and PRKCD knockdown reduced the accumulation of LC3‐II and p62 in SEV‐treated neurons (Figure 2D), indicating restoration of mitophagic degradation. These findings, taken together with the functional mitochondrial recovery observed in Section 3.3, are consistent with improved mitophagic clearance upon PRKCD inhibition, although definitive confirmation of flux directionality would require lysosomal inhibition assays.

### 3.3 PRKCD Distraction Improved SEV‐Mediated Mitochondrial Damage

**FIGURE 3:**
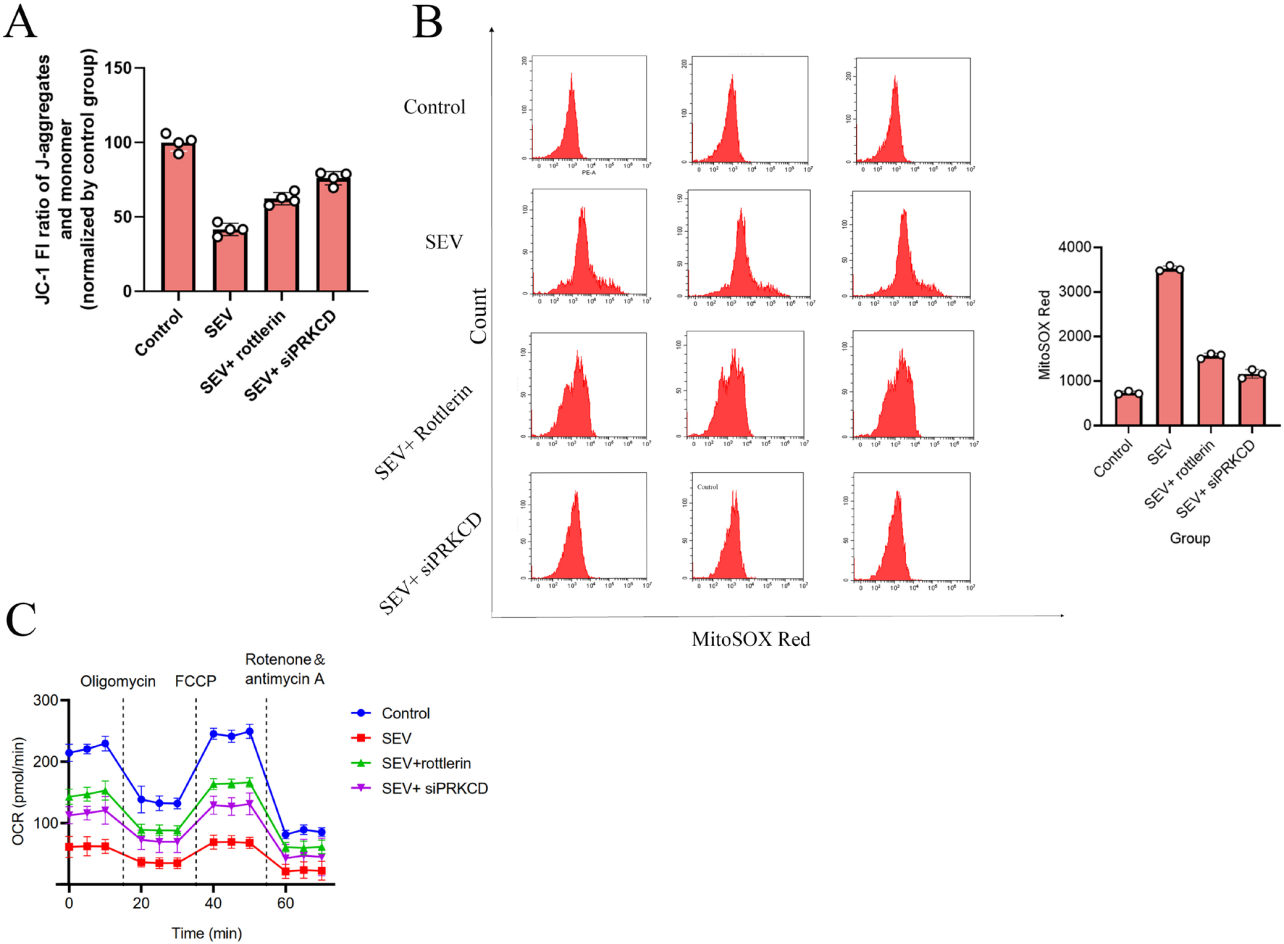
PRKCD inhibition ameliorated SEV‐induced mitochondrial damage. (A) Examination of MMP using MitoProbe JC‐1 by flow cytometry. (B) Measurement of the MitoSOX level by flow cytometry. (C) OCR of neurons using the Seahorse XF‐96 system. *N* = 3.

According to the results from Figure 3A,B, there was an elevation in the MMP and a decrease in the MitoSOX generation after SEV‐induced neurons were treated with rottlerin or PRKCD knockdown, indicating that PRKCD repression enhanced mitochondrial activity and ameliorated mitochondrial damage. Furthermore, the OCR of neurons was declined following the SEV treatment, which was restored with the pharmacological and genic interference of PRKCD (Figure 3C). These data indicate that PRKCD inhibition ameliorates SEV‐induced mitochondrial dysfunction.

### 3.4 PRKCD Modulated Mitophagy Through Interaction With the PINK1/PRKN Axis

**FIGURE 4:**
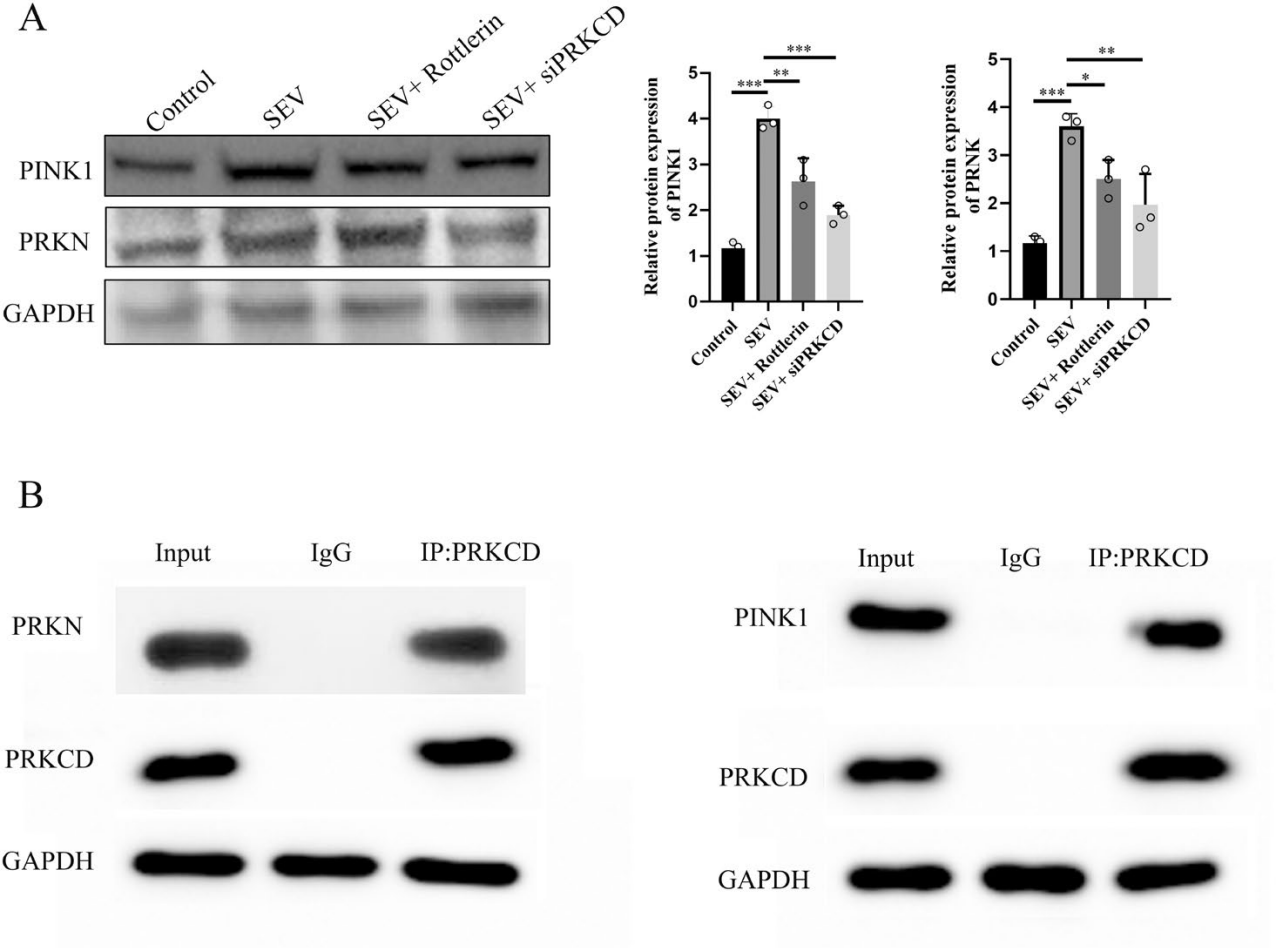
PRKCD‐mediated regulation of PINK1/PRKN mitophagy pathway. (A) Expressions of PINK1 and PRKN by western blot. (B) The interactions between PRKCD and PRKN, as well as PRKCD and PINK1 by Co‐IP. *N* = 3.

To check whether PRKCD modulated mitophagy through interaction with the PINK1/PRKN axis, the expressions of PINK1 and PRKN were examined in neurons. The results exhibited that SEV evoked a noteworthy elevation in the PINK1 and PRKN expressions, which was counteracted with the pharmacological and genic interference of PRKCD (Figure 4A). Co‐immunoprecipitation assays demonstrated physical interactions between PRKCD and PRKN, as well as between PRKCD and PINK1 (Figure 4B). These interactions were detected in both control and SEV‐treated neurons, with increased binding observed following SEV exposure. These findings indicate that PRKCD regulates the PINK1/PRKN mitophagy pathway through direct protein–protein interactions.

### 3.5 PRKCD Mediated Neuronal Mitophagy and Mitochondrial Function by Modulating PRKN

**FIGURE 5:**
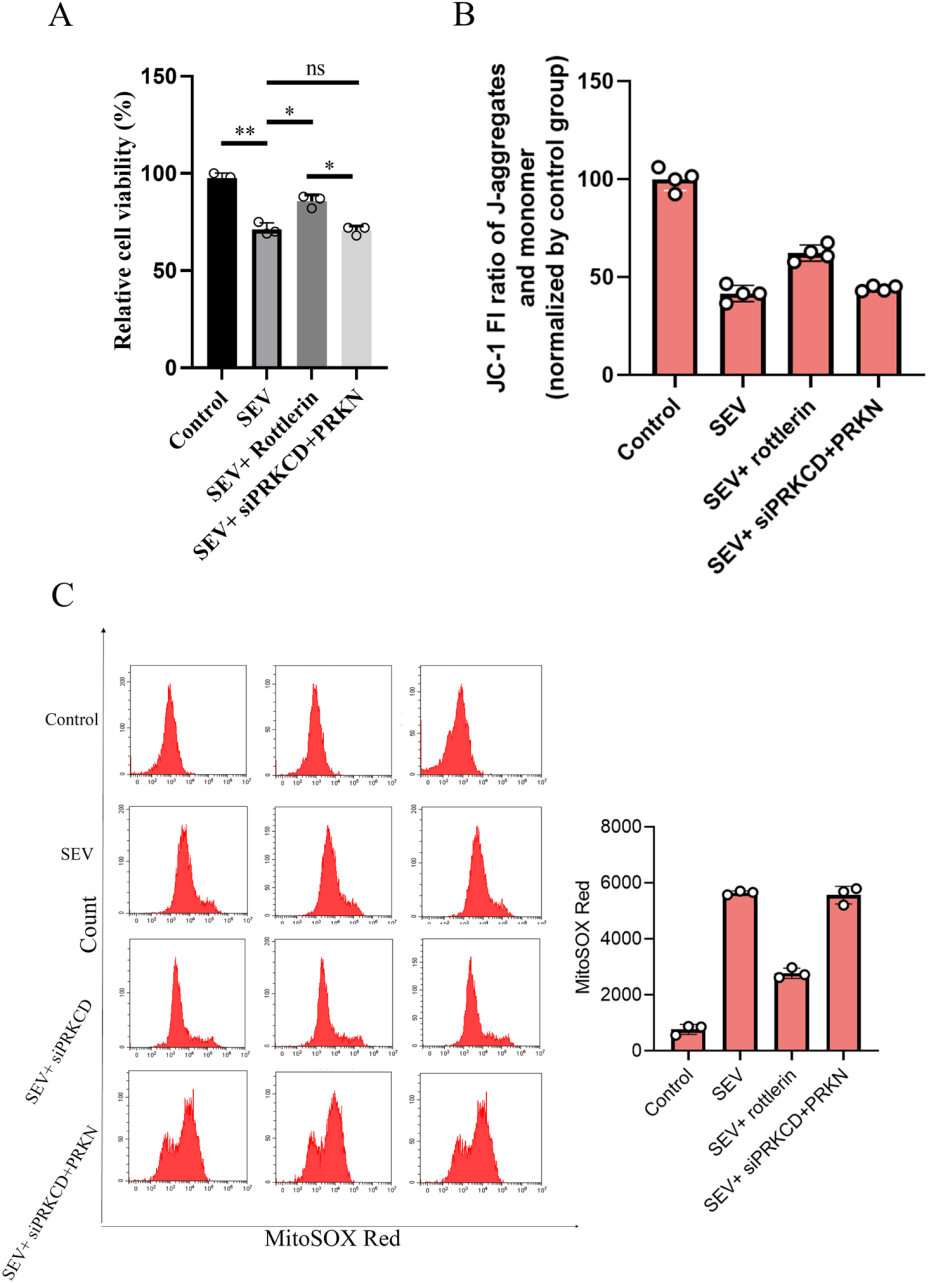
PRKCD modulated neuronal mitophagy and mitochondrial function by modulating PRKN. (A) Neurons were treated with 4.1% SEV for 6 h, and then incubated with the inhibitor of PRKCD, rottlerin for 24 h. Besides, siPRKCD and PRKN overexpressing plasmids were transfected into neurons and then administrated with 4.1% SEV for 6 h. (A) Cell viability by CCK‐8. (B) Examination of MMP using MitoProbe JC‐1 by flow cytometry. (C) Measurement of the MitoSOX level by flow cytometry. *N* = 3.

To further check whether PRKCD functioned on neuronal mitophagy and mitochondrial function by modulating PRKN, the rescue experiments were conducted by overexpressing PRKN in neurons. PRKCD interference recovered the reduction in cell viability and MMP in SEV‐evoked neurons, which was inverted with the PRKN overexpression (Figure 5A,B). However, inverse outcomes were found in the MitoSOX generation (Figure 5C). Collectively, PRKCD regulated neuronal mitophagy and mitochondrial function by modulating PRKN.

### 3.6 PRKCD Knockdown Restored Mitophagic Flux and Improved Cognitive Function in Vivo

**FIGURE 6:**
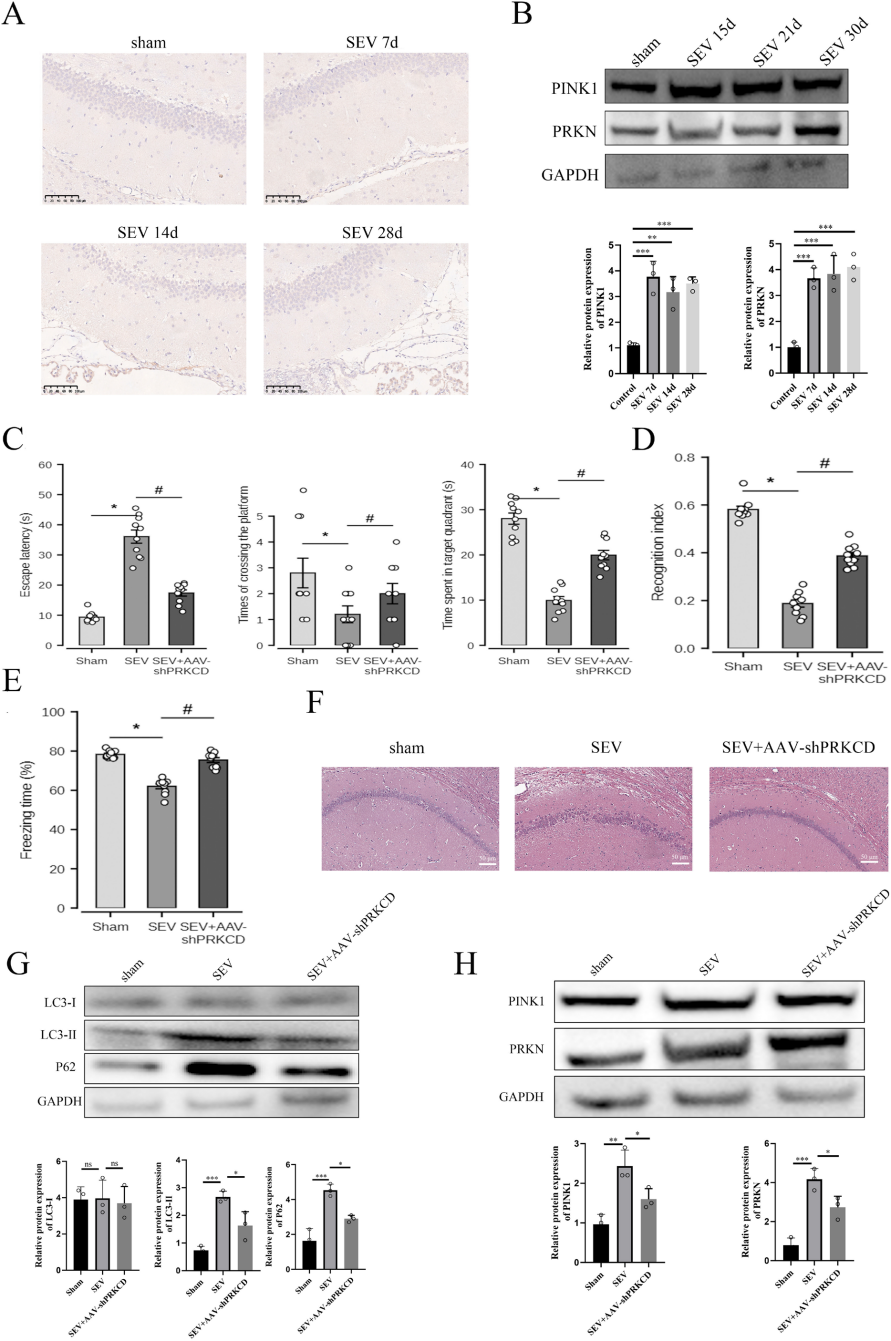
PRKCD knockdown mediated the regulation of PINK1/PRKN mitophagy pathway in vivo. Rats received AAV expressing scrambled shRNA or AAV expressing shRNA targeting PRKCD (5 × 10^12^ viral genomes/mL, 2 μL per side). Fourteen days after AAV injection, rats received a single 4‐h exposure to 2.6% sevoflurane, or humidified 30% O_2_ balanced with N_2_ for the same duration. (A) PRKCD expression by IHC in rat hippocampus at 7, 14 and 28 days post‐exposure with 2.6% sevoflurane for 4 h. *N* = 6. (B) Expression of PINK1 and PRKN by western blot in rats hippocampus at 7, 14 and 28 days post‐exposure. *N* = 6. (C) MWM results 3–7 days post‐exposure, including escape latency, time of crossings the platform and time spent in target quadrant. *N* = 10. (D) Assessment of recognition index by the novel object recognition test 3–7 days post‐exposure. *N* = 10. (E) Evaluation of freezing time by the fear conditioning test 3–7 days post‐exposure. *N* = 10. (F) Pathological examination by HE staining at 7 days post‐exposure. (G) Expressions of LC3‐I, LC3‐II and p62 by western blot at 7 days post‐exposure. *N* = 10. (H) Expression of PINK1 and PRKN by western blot at 7 days post‐exposure. *N* = 10.

To determine the optimal time points for behavioural assessment, we first examined the time course of molecular changes following a single 4‐h exposure to 2.6% sevoflurane. Western blot assessment exhibited that PRKCD, PINK1 and PRKN protein levels were elevated in rat hippocampus at 7, 14 and 28 days post‐exposure compared to sham controls (Figure 6A,B). No significant differences were observed among the three time points. Based on these results, we selected the 7‐day time point for subsequent experiments.

Rats received intrahippocampal injection of AAV‐shPRKCD or control AAV 14 days before SEV exposure, and behavioural testing was conducted 3–7 days post‐exposure. In the MWM test, SEV‐exposed rats exhibited elevated escape latency, fewer platform crossings and less time in the target quadrant than sham controls (Figure 6C). PRKCD knockdown reduced escape latency and increased platform crossings and time in the target quadrant in SEV‐exposed rats (Figure 6C). In the novel object recognition test, SEV exposure decreased the recognition index (Figure 6D), which was restored by PRKCD knockdown (Figure 6D). In the contextual fear conditioning test, SEV exposure reduced freezing time (Figure 6E), and this effect was inverted with PRKCD knockdown (Figure 6E).

Histological examination of the hippocampal CA1 region revealed that sham‐operated rats had normal neuronal morphology with clear nuclear boundaries and visible nucleoli. SEV‐exposed rats displayed neuronal disarrangement, cell shrinkage, nuclear pyknosis and interstitial edema. PRKCD knockdown attenuated these pathological changes in SEV‐exposed rats (Figure 6F). Western blot analysis showed that SEV exposure increased LC3‐II, PINK1 and PRKN expression and elevated p62 levels in the rat hippocampus at 7 days post‐exposure (Figure 6G,H). The concurrent elevation of LC3‐II and p62 indicates impaired mitophagic flux. PRKCD knockdown diminished the aggregation of LC3‐II, p62, PINK1 and PRKN in SEV‐exposed rats (Figure 6G,H), demonstrating restoration of mitophagic flux. These results demonstrate that PRKCD knockdown restores mitophagic flux and ameliorates cognitive deficits in SEV‐induced POCD.

## 4 Discussion

This study showed that PRKCD was expressed in neurons, but not in microglia or astrocytes. SEV administration evoked an upregulation of PRKCD expression. PRKCD interference rescued cell viability of SEV‐treated neurons. Additionally, PRKCD inhibition promoted SEV‐evoked mitophagy and improved mitochondrial damage. Mechanistically, there was an interaction between PRKCD and PRKN and PINK1, and PRKCD interference diminished the expression of these two proteins in SEV‐induced neurons. Thus, overexpression of PRKN counteracted the effect of PRKCD inhibition on mitochondrial damage in SEV‐induced neurons. In vivo, SEV exposure elevated the expressions of PRKCD, PINK1 and PRKN. PRKCD knockdown ameliorated behavioural and pathological defects, and the expression of LC3, p62, PINK1 and PRKN in SEV‐treated rats. In short, PRKCD improved SEV‐induced POCD in aged rats through the regulation of PINK1/PRKN mitophagy pathway.

SEV is a commonly used inhaled anaesthetic in clinical settings, known for its low blood‐gas partition coefficient, rapid absorption and clearance, and minimal irritation [43]. Despite its favourable safety profile, concerns have been raised within the medical community regarding the neurotoxic and cognitive side effects associated with SEV [44, 45]. Previous research has demonstrated that repeated or prolonged exposure to SEV can result in neuronal injury, impede cognitive development, and lead to post‐operative cognitive dysfunction and neurodegenerative diseases [32, 46]. Notably, SEV has been identified as having neurotoxic properties [45, 47]. In this study, neurons and rats were exposed to SEV to simulate POCD. SEV exposure reduced neuronal cell viability in vitro and impaired learning and memory in vivo, indicating the successful establishment of the model. An upregulation of PRKCD expression was observed in both SEV‐treated neurons and managed rats, consistent with findings reported by Lv et al. [31]. This result suggested that PRKCD might play a role in POCD.

The findings from the functional assays illuminated that suppression of PRKCD induced the restoration of MMP and OCR, while also mitigating MitoSOX generation in neurons affected by SEV. This suggested that interference with PRKCD ameliorated mitochondrial damage and enhanced mitochondrial function. Disruption of MMP is recognized as a hallmark of mitophagy [48]. Typically, dysfunctional mitochondria undergo mitophagy to facilitate mitochondrial turnover and maintain homeostasis. Mitochondrial membrane depolarization initiates the aggregation of PINK1 on the OMM, where it phosphorylates and activates Parkin, evoking the ubiquitination of mitochondrial proteins [49]. These ubiquitinated substrates attract autophagy adaptor proteins, such as p62/SQSTM1 and OPTN, which interact with LC3‐II, thereby making the encapsulation of damaged organelles within autophagosomes [50]. Our results indicate that inhibition of PRKCD restored LC3‐II expression and reduced p62 expression in SEV‐induced neurons and rats. These data, in conjunction with the restoration of mitochondrial membrane potential, reduction in mitochondrial ROS, and recovery of oxygen consumption rate, are collectively suggestive of improved mitophagic clearance following PRKCD inhibition. The mitophagy disequilibrium in hippocampal neurons has been identified as a pivotal element in the pathogenesis of POCD. In the context of POCD, disabled mitophagy elicits the persistent assemblage of injured mitochondria, heightening oxidative stress and causing a collapse in energy metabolism. Clinical studies indicate that elderly surgical patients experience an age‐related decline in autophagic clearance capacity, with the extent of this impairment being a strong predictor of the severity of cognitive decline following anaesthesia [51]. Collectively, these findings suggested improved mitochondrial damage and mitophagic clearance following PRKCD interference, while noting that its sufficiency has not been directly demonstrated through overexpression experiments in the present study.

Mechanistically, PRKCD interacted with both PRKN and PINK1, and PRKCD interference reduced the expression of these proteins in neurons subjected to SEV exposure. Moreover, PRKCD knockdown reversed the upregulation of PINK1 and PRKN expression levels in rats exposed to REV. Previous research has shown that the lipid‐binding domains of PRKCD are not essential for mitochondrial recruitment and that cyclin G‐associated kinase (GAK) and PRKCD explicitly manage PRKN‐independent mitophagy, as well as basal mitophagy in vivo [22]. Thus, although a role for PRKCD was demonstrated in PRKN‐independent mitophagy under different experimental conditions [22, 52, 53], our data indicated that, in the context of sevoflurane‐induced POCD, PRKCD functioned through direct protein–protein interactions with PINK1 and PRKN to regulate mitophagic flux. The PRKN overexpression mitigates the effects of PRKCD inhibition on mitochondrial damage in SEV‐exposed neurons. Totally, PRKCD inhibition improved SEV‐induced POCD in aged rats through PRKN‐independent mitophagy.

To summarize, we determined therapeutic role of PRKCD during POCD, and clarified molecular mechanism of PRKCD in facilitating the regulation of PINK1/PRKN mitophagy pathway. Our work offered a promising therapeutic target for POCD. Since POCD is more prevalent among the elderly population, with an incidence rate of 9% to 46% among surgical patients over the age of 65 [3], we chose to conduct the relevant research on aged rats (18 months). This has become the main constraint in promoting the current results to other groups. Thus, the necessity of conducting further validation in young animals, and non‐mammalian models in the future, and factors such as the patient's age should be taken into account for stratification even during the human transformation process. Nevertheless, more mechanistic and functionally related experiments including gain‐of‐function assays are needed in the future to validate PRKCD as effective POCD therapeutic targets. In addition, it should be noted that our assessment of mitophagic flux was based on changes in LC3‐II, p62 and functional mitochondrial parameters without the inclusion of dedicated flux assays such as Bafilomycin A1‐mediated lysosomal inhibition or mitophagy‐specific reporters (e.g., mt‐Keima). Although the concordance of multiple independent lines of evidence supports our interpretation, future studies incorporating these assays would provide more definitive confirmation of mitophagic flux regulation by PRKCD in the context of POCD. A further limitation of this study is the reliance on loss‐of‐function approaches to characterize PRKCD's role in POCD. Although two independent inhibition strategies and a pathway‐specific rescue experiment collectively support the involvement of PRKCD, gain‐of‐function experiments, such as PRKCD overexpression, would be required to establish PRKCD's sufficiency in driving mitochondrial dysfunction and cognitive impairment. Previous findings by Lv et al. [31] demonstrating that PRKCD knockout attenuates sevoflurane‐induced neurotoxicity provide complementary genetic evidence; however, future studies incorporating PRKCD overexpression in both neuronal cultures and aged animal models would further strengthen the mechanistic conclusions. Additionally, in vitro experiments were conducted with *n* = 3 independent biological replicates per condition, which limits the statistical power of the cell‐based analyses. Future studies should employ larger sample sizes to improve the robustness of these findings. Furthermore, formal assessment of normality and variance homogeneity prior to statistical testing was not performed. Although parametric tests are robust to mild violations of their underlying assumptions, future studies with larger sample sizes should incorporate pre‐planned assumption testing to strengthen the statistical rigour of the analyses. In this study, GAPDH was used in western blot experiment as housekeeping gene, but there were not any other housekeeping genes tested. This is one of limitations of this study, which should be confirmed in the future.

## Author Contributions

**Yue Zhang:** conceptualization, investigation, funding acquisition, writing – original draft, writing – review and editing, visualization, validation, methodology, software, formal analysis, project administration, resources, supervision, data curation. **Wei Zhang:** conceptualization, investigation, funding acquisition, writing – original draft, writing – review and editing, visualization, validation, methodology, software, formal analysis, project administration, resources, supervision, data curation. **Yang Yu:** conceptualization, investigation, funding acquisition, writing – original draft, writing – review and editing, visualization, validation, methodology, software, formal analysis, project administration, resources, supervision, data curation. **Pingle Li:** conceptualization, funding acquisition, writing – original draft, investigation, validation, methodology, visualization, writing – review and editing, project administration, formal analysis, data curation, supervision, resources, software. **Yingying Zhao:** conceptualization, investigation, funding acquisition, writing – original draft, writing – review and editing, visualization, validation, methodology, software, formal analysis, project administration, resources, supervision, data curation. **Zepeng He:** conceptualization, investigation, funding acquisition, writing – original draft, writing – review and editing, visualization, validation, methodology, software, formal analysis, resources, supervision, data curation, project administration.

## Funding

This work was supported by Young Scientists Fund of Henan Provincial Natural Science Foundation (242300421489).

## Ethics Statement

The animal experiment procedures were approved by the Animal Care and Use Committee of The First Affiliated Hospital of Zhengzhou University (Approval no. ZZU‐LAC20220909 [1]) and carried out according to the National Institutes of Health Guide for the Care and Use of Laboratory Animals, while also meeting the ARRIVE guidelines.

## Consent

The authors have nothing to report.

## Conflicts of Interest

The authors declare no conflicts of interest.

## Data Availability

The datasets used and/or analysed during the current study are available from the corresponding author on reasonable request.

## References

[1] J. M. Huang , Z. T. Lv , B. Zhang , W. X. Jiang , and M. B. Nie , “Intravenous Parecoxib for Early Postoperative Cognitive Dysfunction in Elderly Patients: Evidence From a Meta‐Analysis,” Expert Review of Clinical Pharmacology 13, no. 4 (2020): 451–460, 10.1080/17512433.2020.1732815. 32077347

[2] M. D. Boone , B. Sites , F. M. von Recklinghausen , A. Mueller , A. H. Taenzer , and S. Shaefi , “Economic Burden of Postoperative Neurocognitive Disorders Among US Medicare Patients,” JAMA Network Open 3, no. 7 (2020): e208931, 10.1001/jamanetworkopen.2020.8931. 32735336 PMC7395237

[3] C. C. Price , C. W. Garvan , and T. G. Monk , “Type and Severity of Cognitive Decline in Older Adults After Noncardiac Surgery,” Anesthesiology 108, no. 1 (2008): 8–17, 10.1097/01.anes.0000296072.02527.18. 18156877 PMC2911011

[4] R. Alalawi and N. Yasmeen , “Postoperative Cognitive Dysfunction in the Elderly: A Review Comparing the Effects of Desflurane and Sevflurane,” Journal of Perianesthesia Nursing 33, no. 5 (2018): 732–740, 10.1016/j.jopan.2017.04.009. 30236581

[5] J. Steinmetz , K. B. Christensen , T. Lund , N. Lohse , and L. S. Rasmussen , “Long‐Term Consequences of Postoperative Cognitive Dysfunction,” Anesthesiology 110, no. 3 (2009): 548–555, 10.1097/ALN.0b013e318195b569. 19225398

[6] J. T. Moller , P. Cluitmans , L. S. Rasmussen , et al., “Long‐Term Postoperative Cognitive Dysfunction in the Elderly ISPOCD1 Study. ISPOCD Investigators. International Study of Post‐Operative Cognitive Dysfunction,” Lancet 351, no. 9106 (1998): 857–861, 10.1016/s0140-6736(97)07382-0. 9525362

[7] X. Zhang , Y. Zhou , M. Xu , and G. Chen , “Autophagy Is Involved in the Sevoflurane Anesthesia‐Induced Cognitive Dysfunction of Aged Rats,” PLoS One 11, no. 4 (2016): e0153505, 10.1371/journal.pone.0153505. 27111854 PMC4844142

[8] C. E. Hughes , T. K. Coody , M. Y. Jeong , J. A. Berg , D. R. Winge , and A. L. Hughes , “Cysteine Toxicity Drives Age‐Related Mitochondrial Decline by Altering Iron Homeostasis,” Cell 180, no. 2 (2020): 296–310.e218, 10.1016/j.cell.2019.12.035. 31978346 PMC7164368

[9] K. He , L. Nie , T. Ali , et al., “Adiponectin Deficiency Accelerates Brain Aging via Mitochondria‐Associated Neuroinflammation,” Immunity & Ageing 20, no. 1 (2023): 15, 10.1186/s12979-023-00339-7. 37005686 PMC10067304

[10] M. Akter , H. Ma , M. Hasan , et al., “Exogenous L‐Lactate Administration in Rat Hippocampus Increases Expression of Key Regulators of Mitochondrial Biogenesis and Antioxidant Defense,” Frontiers in Molecular Neuroscience 16 (2023): 1117146, 10.3389/fnmol.2023.1117146. 37008779 PMC10062455

[11] F. Qu , P. Wang , K. Zhang , et al., “Manipulation of Mitophagy by “All‐In‐One” Nanosensitizer Augments Sonodynamic Glioma Therapy,” Autophagy 16, no. 8 (2020): 1413–1435, 10.1080/15548627.2019.1687210. 31674265 PMC7480814

[12] C. Tang , H. Han , M. Yan , et al., “PINK1‐PRKN/PARK2 Pathway of Mitophagy Is Activated to Protect Against Renal Ischemia‐Reperfusion Injury,” Autophagy 14, no. 5 (2018): 880–897, 10.1080/15548627.2017.1405880. 29172924 PMC6070003

[13] D. A. Kubli and Å. B. Gustafsson , “Mitochondria and Mitophagy: The Yin and Yang of Cell Death Control,” Circulation Research 111, no. 9 (2012): 1208–1221, 10.1161/circresaha.112.265819. 23065344 PMC3538875

[14] Y. Chen , P. Zhang , X. Lin , et al., “Mitophagy Impairment Is Involved in Sevoflurane‐Induced Cognitive Dysfunction in Aged Rats,” Aging (Albany NY) 12, no. 17 (2020): 17235–17256, 10.18632/aging.103673. 32903215 PMC7521530

[15] X. L. Wang , S. T. Feng , Z. Z. Wang , Y. H. Yuan , N. H. Chen , and Y. Zhang , “Parkin, an E3 Ubiquitin Ligase, Plays an Essential Role in Mitochondrial Quality Control in Parkinson's Disease,” Cellular and Molecular Neurobiology 41, no. 7 (2021): 1395–1411, 10.1007/s10571-020-00914-2. 32623547 PMC11448647

[16] D. A. Kubli , X. Zhang , Y. Lee , et al., “Parkin Protein Deficiency Exacerbates Cardiac Injury and Reduces Survival Following Myocardial Infarction,” Journal of Biological Chemistry 288, no. 2 (2013): 915–926, 10.1074/jbc.M112.411363. 23152496 PMC3543040

[17] J. A. Williams , H. M. Ni , Y. Ding , and W. X. Ding , “Parkin Regulates Mitophagy and Mitochondrial Function to Protect Against Alcohol‐Induced Liver Injury and Steatosis in Mice,” American Journal of Physiology. Gastrointestinal and Liver Physiology 309, no. 5 (2015): G324–G340, 10.1152/ajpgi.00108.2015. 26159696 PMC4556950

[18] R. K. Bruick , “Expression of the Gene Encoding the Proapoptotic Nip3 Protein Is Induced by Hypoxia,” Proceedings of the National Academy of Sciences of the United States of America 97, no. 16 (2000): 9082–9087, 10.1073/pnas.97.16.9082. 10922063 PMC16825

[19] G. F. Allen , R. Toth , J. James , and I. G. Ganley , “Loss of Iron Triggers PINK1/Parkin‐Independent Mitophagy,” EMBO Reports 14, no. 12 (2013): 1127–1135, 10.1038/embor.2013.168. 24176932 PMC3981094

[20] J. F. Zhao , C. E. Rodger , G. F. G. Allen , S. Weidlich , and I. G. Ganley , “HIF1α‐Dependent Mitophagy Facilitates Cardiomyoblast Differentiation,” Cell Stress 4, no. 5 (2020): 99–113, 10.15698/cst2020.05.220. 32420530 PMC7212530

[21] P. Jaakkola , D. R. Mole , Y. M. Tian , et al., “Targeting of HIF‐Alpha to the von Hippel‐Lindau Ubiquitylation Complex by O2‐Regulated Prolyl Hydroxylation,” Science 292, no. 5516 (2001): 468–472, 10.1126/science.1059796. 11292861

[22] M. J. Munson , B. J. Mathai , M. Y. W. Ng , et al., “GAK and PRKCD Are Positive Regulators of PRKN‐Independent Mitophagy,” Nature Communications 12, no. 1 (2021): 6101, 10.1038/s41467-021-26331-7. PMC8528926 34671015

[23] Y. Ono , T. Fujii , K. Ogita , U. Kikkawa , K. Igarashi , and Y. Nishizuka , “The Structure, Expression, and Properties of Additional Members of the Protein Kinase C Family,” Journal of Biological Chemistry 263, no. 14 (1988): 6927–6932. 2834397

[24] M. E. Reyland and D. N. Jones , “Multifunctional Roles of PKCδ: Opportunities for Targeted Therapy in Human Disease,” Pharmacology & Therapeutics 165 (2016): 1–13, 10.1016/j.pharmthera.2016.05.001. 27179744 PMC5116389

[25] X. Chen , R. Kong , Y. Qi , et al., “MiR‐26b‐5p Mediates Radioresistance and Immunosuppression via Targeting PRKCD in Non‐Small Cell Lung Cancer,” Journal of Cancer Research and Clinical Oncology 151, no. 10 (2025): 262, 10.1007/s00432-025-06310-x. 40965741 PMC12446157

[26] S. Zafar , Y. Badshah , M. Shabbir , et al., “Missense Variants in PRKCD: Elucidating Their Potential Association With Breast Cancer,” Breast Cancer Research 27, no. 1 (2025): 139, 10.1186/s13058-025-02090-x. 40722182 PMC12305980

[27] R. Feng , Z. Fang , S. Zhao , et al., “SIRT6 Lysine‐Demyristoylates ATF2 to Ameliorate Vascular Injury via PRKCD/VE‐Cadherin Pathway Regulating Vascular Endothelial Barrier,” Advanced Science 12 (2025): e04948, 10.1002/advs.202504948. 40810740 PMC12591191

[28] R. Yadav , N. Nambiar , M. Shah , and B. D. Patel , “Exploring the Therapeutic Potential of *Momordica charantia* in Targeting Protein Kinase C Delta (PRKCD) for Type 2 Diabetes Mellitus: Insights From Network Pharmacology, Molecular Docking, and Molecular Dynamics Simulations,” In Silico Pharmacology 13, no. 2 (2025): 99, 10.1007/s40203-025-00385-7. 40636081 PMC12234958

[29] J. Zhou , Y. Xu , H. Wang , K. Wang , and C. Chen , “Unlocking New Treatment Horizons for Celiac Disease: PRKCD Revealed as a Promising Target Through Mendelian Randomization,” Endocrine, Metabolic & Immune Disorders Drug Targets 26 (2025): e18715303410531, 10.2174/0118715303410531250815124524. PMC13284647 40910294

[30] X. Guo , L. Liu , J. Luo , et al., “Constraint‐Induced Movement Therapy Combined With Mesenchymal Stem Cell Transplantation Promotes Myelination and Functional Recovery by Inhibiting PRKCD/MEK/ERK Pathway in Hemiplegic Cerebral Palsy Rats,” Stem Cell Res Ther 16, no. 1 (2025): 447, 10.1186/s13287-025-04544-7. 40841922 PMC12369169

[31] T. Lv , F. Jia , G. Wang , et al., “Sevoflurane Causes Neurotoxicity and Cognitive Impairment by Regulating Hippo Signaling Pathway‐Mediated Ferroptosis via Upregulating PRKCD,” Experimental Neurology 377 (2024): 114804, 10.1016/j.expneurol.2024.114804. 38704083

[32] X. Tang , Y. Zhao , Z. Zhou , et al., “Resveratrol Mitigates Sevoflurane‐Induced Neurotoxicity by the SIRT1‐Dependent Regulation of BDNF Expression in Developing Mice,” Oxidative Medicine and Cellular Longevity 2020 (2020): 9018624, 10.1155/2020/9018624. 32148659 PMC7049870

[33] C. Luchena , J. Zuazo‐Ibarra , J. Valero , C. Matute , E. Alberdi , and E. Capetillo‐Zarate , “A Neuron, Microglia, and Astrocyte Triple co‐Culture Model to Study Alzheimer's Disease,” Frontiers in Aging Neuroscience 14 (2022): 844534, 10.3389/fnagi.2022.844534. 35493929 PMC9048896

[34] M. Y. Kim , K. Lee , H. I. Shin , and D. Jeong , “Specific Targeting of PKCδ Suppresses Osteoclast Differentiation by Accelerating Proteolysis of Membrane‐Bound Macrophage Colony‐Stimulating Factor Receptor,” Scientific Reports 9, no. 1 (2019): 7044, 10.1038/s41598-019-43501-2. 31065073 PMC6504882

[35] D. Li , L. Liu , L. Li , et al., “Sevoflurane Induces Exaggerated and Persistent Cognitive Decline in a Type II Diabetic Rat Model by Aggregating Hippocampal Inflammation,” Frontiers in Pharmacology 8 (2017): 886, 10.3389/fphar.2017.00886. 29238302 PMC5712596

[36] P. Dong , J. Zhao , N. Li , et al., “Sevoflurane Exaggerates Cognitive Decline in a Rat Model of Chronic Intermittent Hypoxia by Aggravating Microglia‐Mediated Neuroinflammation via Downregulation of PPAR‐γ in the Hippocampus,” Behavioural Brain Research 347 (2018): 325–331, 10.1016/j.bbr.2018.03.031. 29574103

[37] N. M. Razali and Y. B. Wah , “Power comparisons of Shapiro‐Wilk, Kolmogorov‐Smirnov, Lilliefors and Anderson‐Darling tests,” (2011).

[38] Z. Li , H. Qi , L. Na , et al., “HMGB1 Promotes LPS‐Induced M1 Polarization and Apoptosis in Microglia by Mediating the Expression of Immune and Inflammation‐Related Genes,” Brain Research Bulletin 233 (2025): 111615, 10.1016/j.brainresbull.2025.111615. 41187887

[39] S. Deng , G. Mu , J. Li , X. Yu , Q. Li , and B. Lu , “METTL14‐Mediated m6A Modification of DUSP6 mRNA Participating in Postoperative Cognitive Dysfunction due to Sevoflurane Anesthesia,” Journal of Physiological Sciences 75, no. 3 (2025): 100048, 10.1016/j.jphyss.2025.100048. PMC12617634 41175640

[40] X. Li , W. Ding , X. Zhang , et al., “Caveolin‐1‐Mediated Blood‐Brain Barrier Disruption via MMP2/9 Contributes to Postoperative Cognitive Dysfunction,” Neurochemical Research 50, no. 4 (2025): 210, 10.1007/s11064-025-04458-z. 40571858 PMC12202573

[41] X. Li , X. Li , Q. Zhang , et al., “Prostaglandin Endoperoxide Synthase 2 Regulates Neuroinflammation to Mediate Postoperative Cognitive Dysfunction in Mice,” Scientific Reports 15, no. 1 (2025): 17355, 10.1038/s41598-025-01121-z. 40389478 PMC12089394

[42] Z. Qi , L. Ding , Y. Zhao , et al., “Astrocytic EAAT1 Suppression by EV‐ACLY Underlies Glutamate Imbalance and Cognitive Impairment in POCD,” Communications Biology 9 (2026): 636, 10.1038/s42003-026-09888-1. 41857316 PMC13168382

[43] Y. Wang , X. X. Ming , and C. P. Zhang , “Fluorine‐Containing Inhalation Anesthetics: Chemistry, Properties and Pharmacology,” Current Medicinal Chemistry 27, no. 33 (2020): 5599–5652, 10.2174/0929867326666191003155703. 31580246

[44] C. Apai , R. Shah , K. Tran , and S. Pandya Shah , “Anesthesia and the Developing Brain: A Review of Sevoflurane‐Induced Neurotoxicity in Pediatric Populations,” Clinical Therapeutics 43, no. 4 (2021): 762–778, 10.1016/j.clinthera.2021.01.024. 33674065

[45] M. Sun , Z. Xie , J. Zhang , and Y. Leng , “Mechanistic Insight Into Sevoflurane‐Associated Developmental Neurotoxicity,” Cell Biology and Toxicology 38, no. 6 (2022): 927–943, 10.1007/s10565-021-09677-y. 34766256 PMC9750936

[46] C. Huang , J. M. T. Chu , Y. Liu , V. S. W. Kwong , R. C. C. Chang , and G. T. C. Wong , “Sevoflurane Induces Neurotoxicity in the Animal Model With Alzheimer's Disease Neuropathology via Modulating Glutamate Transporter and Neuronal Apoptosis,” International Journal of Molecular Sciences 23, no. 11 (2022): 6250, 10.3390/ijms23116250. 35682930 PMC9181124

[47] Y. F. Bai , W. J. Li , Y. W. Ji , L. X. An , L. Zhang , and J. F. Li , “Sevoflurane Induces Neurotoxic Effects on Developing Neurons Through the WNK1/NKCC1/Ca2+ /Drp‐1 Signalling Pathway,” Clinical and Experimental Pharmacology & Physiology 50, no. 5 (2023): 393–402, 10.1111/1440-1681.13755. 36733226

[48] M. Onishi , K. Yamano , M. Sato , N. Matsuda , and K. Okamoto , “Molecular Mechanisms and Physiological Functions of Mitophagy,” EMBO Journal 40, no. 3 (2021): e104705, 10.15252/embj.2020104705. 33438778 PMC7849173

[49] D. P. Narendra , S. M. Jin , A. Tanaka , et al., “PINK1 Is Selectively Stabilized on Impaired Mitochondria to Activate Parkin,” PLoS Biology 8, no. 1 (2010): e1000298, 10.1371/journal.pbio.1000298. 20126261 PMC2811155

[50] S. A. Sarraf , M. Raman , V. Guarani‐Pereira , et al., “Landscape of the PARKIN‐Dependent Ubiquitylome in Response to Mitochondrial Depolarization,” Nature 496, no. 7445 (2013): 372–376, 10.1038/nature12043. 23503661 PMC3641819

[51] N. Sun , J. Yun , J. Liu , et al., “Measuring in Vivo Mitophagy,” Molecular Cell 60, no. 4 (2015): 685–696, 10.1016/j.molcel.2015.10.009. 26549682 PMC4656081

[52] Y. Lei and D. J. Klionsky , “New Regulators of PRKN‐Independent Mitophagy,” Autophagy 18, no. 1 (2022): 1–3, 10.1080/15548627.2021.2012867. 34927553 PMC8865297

[53] M. J. Munson , B. J. Mathai , M. Y. W. Ng , L. Trachsel‐Moncho , L. R. de la Ballina , and A. Simonsen , “GAK and PRKCD kinases regulate basal mitophagy,” Autophagy 18, no. 2 (2022): 467–469, 10.1080/15548627.2021.2015154. 35001811 PMC8942551

